# ADAPTING COGNITIVE REMEDIATION GROUP THERAPY AS A HYBRID INTERVENTION FOR PEOPLE WITH HIV AND COGNITIVE CONCERNS

**DOI:** 10.1093/geroni/igad104.1686

**Published:** 2023-12-21

**Authors:** Andrew Eaton, Jenny Hui, Marvelous Muchenje, Kate Murzin, Soo Chan Carusone, francisco Ibáñez-Carrasco, Nuelle Novik, Sharon Walmsley

**Affiliations:** University of Regina, Saskatoon, Saskatchewan, Canada; University of Toronto, Toronto, Ontario, Canada; University of Toronto, Toronto, Ontario, Canada; Realize, Toronto, Ontario, Canada; McMaster University, Hamilton, Ontario, Canada; University of Toronto, Toronto, Ontario, Canada; University of Regina, Regina, Saskatchewan, Canada; University Health Network, Toronto, Ontario, Canada

## Abstract

Cognitive impairment is a significant health issue for people aging with HIV/AIDS. With pharmacological treatment lacking, psychosocial group therapies may best help people aging with HIV and cognitive challenges cope with symptoms. The COVID-19 pandemic demonstrated how in-person group therapies need adaptation for hybrid or online delivery. Peer-led focus groups discussed adapting cognitive remediation group therapy (CRGT) as a hybrid or online intervention. CRGT combines mindfulness-based stress reduction and brain training activities. Purposive sampling recruited people aging with HIV (40+) who self-identified cognitive concerns and resided in two Canadian provinces. Content analysis was employed on transcripts by 7 independent coders. Ten, two-hour focus groups were conducted between August and November 2022. Participant (n=45) demographics included age (M=53.22, SD=7.62) gender (45% women, 42% men, 13% trans/non-binary), sexuality (42% gay, 40% heterosexual, 18% other), ethnicity (45% white, 33% black, 13% Indigenous, 9% mixed-race), and employment (33% employed, 67% retired/disability), and all were retained in care. Overall, participants responded favourably to CRGT’s modalities and preferred a hybrid model blending in-person and online interactions. Preferred intervention facilitators were peers and mental health professionals. Knowledge of HIV’s impacts on cognitive health, including HIV-associated neurocognitive disorder, was very low despite high reports of cognitive concerns (e.g., trouble remembering, impaired attention, difficulty problem-solving). Given the aging of the HIV population in Canada and the United States, increasing support will be required to improve quality of life. This presentation will discuss a hybrid CRGT adaptation, alongside considerations for how COVID-19 has impacted intervention research.

